# Translation termination depends on the sequential ribosomal entry of eRF1 and eRF3

**DOI:** 10.1093/nar/gkz177

**Published:** 2019-03-15

**Authors:** Christian Beißel, Bettina Neumann, Simon Uhse, Irene Hampe, Prajwal Karki, Heike Krebber

**Affiliations:** 1 Abteilung für Molekulare Genetik, Institut für Mikrobiologie und Genetik, Göttinger Zentrum für Molekulare Biowissenschaften (GZMB), Georg-August Universität Göttingen, Germany; 2 Department of Physical Biochemistry, Max Planck Institute for Biophysical Chemistry, Göttingen, Germany

## Abstract

Translation termination requires eRF1 and eRF3 for polypeptide- and tRNA-release on stop codons. Additionally, Dbp5/DDX19 and Rli1/ABCE1 are required; however, their function in this process is currently unknown. Using a combination of *in vivo* and *in vitro* experiments, we show that they regulate a stepwise assembly of the termination complex. Rli1 and eRF3-GDP associate with the ribosome first. Subsequently, Dbp5-ATP delivers eRF1 to the stop codon and in this way prevents a premature access of eRF3. Dbp5 dissociates upon placing eRF1 through ATP-hydrolysis. This in turn enables eRF1 to contact eRF3, as the binding of Dbp5 and eRF3 to eRF1 is mutually exclusive. Defects in the Dbp5-guided eRF1 delivery lead to premature contact and premature dissociation of eRF1 and eRF3 from the ribosome and to subsequent stop codon readthrough. Thus, the stepwise Dbp5-controlled termination complex assembly is essential for regular translation termination events. Our data furthermore suggest a possible role of Dbp5/DDX19 in alternative translation termination events, such as during stress response or in developmental processes, which classifies the helicase as a potential drug target for nonsense suppression therapy to treat cancer and neurodegenerative diseases.

## INTRODUCTION

When a ribosome arrives at a stop codon on the mRNA, protein synthesis is terminated and the peptide is released (1). In eukaryotes, two essential release factors are well known to mediate translation termination. The eukaryotic release factor 1 (eRF1), in *Saccharomyces cerevisiae* encoded by *SUP45*, is the only class I termination factor in eukaryotes that recognizes all three different stop codons (UAG, UAA, UGA) and subsequently mediates the hydrolysis of the peptidyl-tRNA in the ribosomal peptidyl-transferase center (PTC). In addition, the class II eukaryotic release factor 3 (eRF3), in *S. cerevisiae* encoded by *SUP35*, enhances translation termination efficiency with its GTPase activity (2,3). Most termination models (Supplementary Figure S1A) anticipate that eRF1 and eRF3–GTP enter the ribosome together as a ternary complex once the stop codon is reached (1,2,4), as both factors strongly interact with each other via their C-terminal domains (5,6). Supposedly, successful stop codon recognition by eRF1 induces GTP-hydrolysis of eRF3, which in turn leads to a conformational rearrangement in eRF1 resulting in its active form, which positions its GGQ-motif in the PTC and mediates hydrolysis of the ester bond of the peptidyl-tRNA (7–9).

In light of these mostly *in vitro* studies, nothing seems to be missing; however, novel factors essential for translation termination *in vivo* were discovered and need to be incorporated into a comprehensive model: The DEAD-box RNA helicase Dbp5, encoded by *RAT8* (human DDX19) (10), its stimulating co-factors Gle1 plus inositol hexakisphosphate IP_6_ (11,12), the iron-sulfur containing ATP-binding cassette protein Rli1 (human ABCE1) (13,14) and the initiation factor eIF3, including Hcr1 (15). Dbp5 and Gle1 are well known for their function in mRNA-export through nuclear pore complexes (NPCs) (16). Using its regulated ATPase cycle, Dbp5 remodels RNA–protein complexes at the cytoplasmic side of the NPC on emerging mRNAs (17). By dissociation of the export receptor Mex67-Mtr2 (human TAP-p15) from the arriving mRNAs, its backsliding is prevented and directionality of the transport event established. Its co-factors Gle1 and IP_6_ stimulate ATP-hydrolysis leading to RNP-release and binding of Dbp5-ADP to the NPC-protein Nup159 (human Nup214). Importantly, this binding leads to ADP-release, a conformational change and the binding of ATP (16,17). The ATPase activity of Dbp5 is also essential for efficient translation termination (10,12). In addition to these functions, Dbp5 plays also a role in the export of both ribosomal subunits (18). However, in contrast, to mRNA export and translation termination, Dbp5 acts independently of its ATPase activity in ribosome export (18).

Rli1 functions in biogenesis and nuclear export of pre-ribosomal subunits (19–21), translation initiation (22), termination (13) and in particular in ribosome recycling (23). Rli1 is a soluble member of the ATP-binding cassette (ABC) protein superfamily that contains two nucleotide-binding domains (NBDs) and two N-terminal iron-sulfur clusters. A hinge domain connects both NBDs forming a cleft, which is open in the ADP-bound state, while ATP-binding induces its closure with a concomitant movement of the iron-sulfur domain allowing ATP-hydrolysis. This ATP-dependent tweezers-like motion converts chemical energy into mechanical power, which is important for splitting the ribosome into its ribosomal subunits (24). The protein is highly conserved in eukaryotes and essential in all organisms tested (22). Interestingly, Rli1 acts ATP-hydrolysis independent during translation termination (13,25). It was suggested that Rli1 associates with the termination complex upon dissociation of eRF3–GDP, taking over its position to keep eRF1 in its favourable position to facilitate peptidyl-tRNA hydrolysis (4,26).

The initiation factor eIF3 has recently been associated with translation termination, because mutations in its subunits reduce the rate of stop codon readthrough (15). Interestingly, deletion of the substoichiometric component *HCR1* shows an increased readthrough activity and this phenotype was suppressed by high copy *RLI1*. A model was proposed in which Hcr1 is not a bona fide translation initiation factor, but rather acts in termination by promoting GDP–eRF3 ejection from the ribosomes (15).

So far, no translation termination model is available that includes all of these factors that support termination and many results were obtained from *in vitro* assays with purified components. Therefore, we analysed the process in *S. cerevisiae in vivo* and *in vitro* with all participating factors and uncovered a sequential recruitment mechanism, in which Rli1 and eRF3 wait at the ribosome for the entry of Dbp5 that delivers eRF1 and at the same time shields it from premature access of eRF3. Upon proper positioning Dbp5 dissociates, allowing eRF3 to contact and stimulate eRF1 activity. This stepwise entry of the termination factors and in particular the Dbp5 controlled eRF1–eRF3 interaction, prevents premature and inefficient translation termination.

## MATERIALS AND METHODS

### Yeast strains and plasmids

All *S. cerevisiae* strains, plasmids and oligonucleotides used in this study are listed in the Expanded View Supplementary Tables S1, S2 and S3, respectively. For growth analyses, cells were spotted in 10-fold serial dilutions onto selective agar plates and grown for 3 days at the indicated temperatures.

The strains, HKY1622 and HKY1623, were generated by crossing HKY1271 (*RLI1-GFP*) and HKY446 (*sup45-2*). Crossing the strains HKY445 (WT of HKY446) or HKY446 with the strain HKY1122 (Prt1-GFP) produced the strain HKY1915 and the corresponding WT HKY1914. The strain HKY1921 was generated by crossing the strains HKY477 (*rat8-2-myc*) and HKY1907 (*trp5Δ*) and exchange of pHK629 with pHK693. For the generation of plasmid pHK1292, the GFP ORF was amplified by polymerase chain reaction using the primers HK1194 and HK1195 and inserted via Xh*o*I and PstI sites into pHK887 (*2μ Rli1-HA LEU2*) replacing the HA-tag. To create pHK1474 and pHK1475, the *RLI1-GFP* ORF with promoter and terminator was amplified from pHK1292 with the primers HK2136 and HK2137 and inserted via Gibson assembly reaction into pHK86 and pHK87, respectively, which were linearized by SacI and SalI digestion. The *GLE1* ORF was amplified from gDNA with HK1398 and HK1399 and inserted via BamHI site into pHK825 (*CEN P_ADH1_3xMYC URA3*) to generate pHK1323. For the generation of pHK1283, the *SUP35* ORF was amplified with the primers HK1109 and HK1110 and inserted via EcoRI and XhoI sites into pGEX-4T-1. The *SUP45* ORF was amplified with the primers HK1144 and HK1156 and inserted via NdeI and XhoI sites into pET28a to create pHK1280. The *SUP45Δ1237-1311* ORF was amplified with the primers HK1146 and HK1147 and inserted via BamHI and XhoI sites into pET15b to generate pHK1278. To generate pHK1394, the *GLE1* ORF was amplified with the primers HK1613 and HK1614 and inserted via Gibson assembly reaction into pETMBP1_1a that was linearized by SacI and NcoI digestion.

### Co-immunoprecipitation experiments

*In vivo* interactions studies were carried out following the protocols published previously (18,27). For immunoprecipitation of GFP-tagged proteins, 10 μl slurry of GFP-Trap_A beads (Chromotek) and for TAP-tagged proteins, 20 μl slurry of IgG-Sepharaose beads (GE Healthcare) were used per reaction and incubated with 200 (high abundant proteins) and up to 2000 μl (for low abundant proteins) of the clarified lysate for 3 h rotating at 4°C. If indicated, the samples were treated with 0.2 mg/ml RNase A (AppliChem) for additional 30 min at 4°C. Finally, the eluted proteins were separated on 10% SDS-polyacrylamide gels and analysed by western blotting.

### Sucrose-density gradient fractionation

The experiments were essentially performed as described previously (18) with the following modifications. For elongation factor mutants, no cycloheximide treatment was performed meaning that cells were directly harvested upon 1 h temperature shift, cycloheximide was omitted from the lysis buffer. For protein analyses, 15 OD_260nm_ units of lysates were loaded onto the top of linear 7–47% (w/v) sucrose gradients and centrifuged for 2 h and 40 min at 40 000 rpm and 4°C in a TH-641 rotor and Sorvall WX80 ultracentrifuge (Thermo Scientific). After gradient fractionation, protein fractions were precipitated with 10% trichloroacetic acid, washed twice with 80% aceton and subjected to sodium dodecylsulphate-polyacrylamide gel electrophoresis (SDS-PAGE) and western blotting.

### Western blot analyses and quantification

Polyclonal rabbit antibodies against Dbp5 (dilution 1:1000), eRF1 and eRF3 (kindly provided by D. Bedwell, dilution for both 1:1000), uL29 = Rpl35 and uS3 = Rps3 (kindly provided by M. Seedorf, dilution 1:5000 and 1:10 000 respectively, or anti-Rps3 peptide antibody 1:500), Aco1, Hem15, Por1 and Zwf1 (kindly provided by R. Lill, dilution 1:1000, 1:7000, 1:2000, and 1:4000, respectively), Asc1 (kindly provided by G. Braus, dilution 1:2000) and Cdc28 (sc-28550; Santa Cruz, dilution 1:2000) were used. GFP-tagged proteins were detected with anti-GFP antibodies (sc-8334; Santa Cruz, dilution 1:1000), or GF28R (Pierce Protein Biology, 1:5000), or (ab183734; Abcam; 1:10 000), MYC-tagged proteins with an anti-MYC antibody (sc-789; Santa Cruz, dilution 1:750) and HA-tagged proteins with an anti-HA antibody (sc-57592 and sc-7392; Santa Cruz, dilution 1:750) and GST-tagged proteins with an anti-GST antibody (sc-138; Santa Cruz, dilution 1:2000). Secondary anti-rabbit IgG (H+L)-HRPO and anti-mouse IgG (H+L)-HRPO (Dianova) antibodies were used and detected with Amersham ECL Prime Western Blotting Detection Reagent (GE Healthcare) or WesternBright Chemilumineszenz Substrat Quantum (Biozym) and the FUSION-SL chemiluminescence detection system (Peqlab). Quantification of western blot signals was performed with the Bio1D software (Peqlab) or with ImageStudio Lite (Li-COR Biosciences). For statistical analyses of co-immunoprecipitation studies, the intensity of co-precipitated bands was related to that of the pull-down and finally, the ratio of the mutant or treated strains was compared to the wild typical ratio. For quantification of the sucrose density gradient fractionation experiments, the intensity of each fraction was measured and the polysomal fractions were compared to the sum of the 80S, 60S, 40S and non-ribosomal fractions.

### *In vitro* binding studies

GST-Dbp5, GST-eRF1 and GST-eRF3ΔN65, a more stable version of eRF3 that has a truncated C-terminus, were expressed in *Escherichia coli* Rosetta 2 cells, purified by affinity chromatography with GSTrap 4B Glutathione Sepharose (GE Healthcare) and stored at −80°C in elution buffer (for GST-Dbp5: 50 mM Tris pH 7.5, 150 mM NaCl, 30 mM Glutathione reduced) (for GST-eRF1 and GST-eRF3: 20 mM HEPES pH 7.5, 500 mM NaCl, 5% glycerol, 4 mM β-MeOH, 30 mM Glutathione reduced). To obtain untagged Dbp5, eRF1 and eRF3, the purified GST-tagged proteins were cleaved with PreScission protease overnight at 4°C. Afterwards, GST was removed by performing a second affinity chromatography with GSTrap 4B Glutathione Sepharose (GE Healthcare). The proteins were further purified by running the collected flow through over a gel filtration chromatography. Purified GST–Dbp5 and Dbp5 were stored in 50 mM Tris pH 7.5, 150 mM NaCl, 10% glycerol at −80°C, whereas GST-eRF1, eRF1, GST- eRF3 and eRF3 were stored in 50 mM NaCl pH 7.5, 20 mM HEPES, 5% glycerol and 4 mM β-MeOH at 80°C.

For binding studies either purified proteins as indicated above were used or Rosetta II (DE3) cells were transformed with pET15b-HIS_6_-SUP45 delta 25 (eRF1 lacking 25aa at the C-terminus), pET28a-HIS_6_-SUP45, pGEX4T1-GST-SUP35, pGEX-6P-1-GST-RLI1 and pGEX6P1-GST. Overexpression was induced by growing the cells for 3 days in auto-inducing media (LB media with 0.5% (v/v) glycerol, 0.05% (v/v) Ggucose and 0.2% (v/v) lactose plus 25 mM K_2_HPO_4_, 25 mM NaH_2_PO_4_, 50 mM NH_4_Cl and 0.5 mM Na_2_SO_4_) at 16°C. Cells were harvested and resuspended in lysis buffer (50 mM Tris–HCl, pH 7.5, 150 mM NaCl, 2 mM MgCl, 5% (v/v) glycerol, 1 mM Dithiothreitol (DTT), 0.2% (v/v) NP-40 and ethylenediaminetetraacetic acid (EDTA)-free protease inhibitor mix (Roche)). After cell lysis by sonication and centrifugation, the supernatant was used for further analysis. The used GSTrap 4B Glutathione Sepharose (GE Healthcare) beads were pre-incubated with 3% Albumin Fraction V (Roche) and mixed with lysis buffer. Next, the GST tagged proteins or only GST were incubated with 15 μl slurry of GSTrap for 2 h at 4°C. After several washing steps with buffer (excluding protease inhibitor), the beads were incubated with the protein lysates for additional 2 h at 4°C. For the competition assay, the eRF1 lysate was first incubated with GST-Dbp5 bound to the GSTrap for 20 min at 4°C, before adding purified eRF3ΔN65 in the indicated amounts and further incubation for 1 h at 4°C. After five washing steps with lysis buffer, the proteins that were bound to the beads were analysed by SDS-PAGE and western blot.

Prt1-GFP and Nip1-GFP were purified from yeast cells before the *in vitro* binding assay was carried out with recombinantly expressed GST-Rli1.

### Readthrough assay

The dual reporter β-galactosidase luciferase assay was basically performed as described previously (10,28). Briefly, all analysed yeast strains were transformed with the reporter plasmids pHK607 or pHK608, respectively. Yeast cells were grown at 25°C to mid-log phase, shifted for 30 min to 37°C and afterward divided: 20 OD_600_ were used for the luciferase assay and 50 ml that were split for triplicates for the β-galactosidase assay,

The luciferase assay was performed with the ‘Beetle-Juice Luciferase Assay Firefly’ kit (p.j.k GmbH) according to the manufacturer’s protocol. For that, cell pellets were lysed with 300 μl glass beads in a FastPrep-24 machine (MP Biomedical) in 400 μl lysis buffer (77 mM K_2_HPO_4_, 23 mM KH_2_PO_4_, 0.2% Triton X-100, 1 mM DTT) supplemented with Complete, EDTA-free protease inhibitor cocktail (Roche). The lysates were centrifuged twice for 5 min at 21 000 × *g* and 4°C. Afterwards, 20 μl of the cleared lysate was transferred in triplicate into a white 96-well plate, supplemented with always 50 μl substrate and measured with the luminometer (Victor X3 2030 multilabel reader from Perkin Elmer).

For the β-galactosidase assay, all cell pellets were resuspended in 300 μl of Z-buffer (100 mM phosphate buffer pH 7.0, 10 mM KCl, 1 mM MgSO_4_) and lysed with the freeze-and-thaw method (1 min in liquid nitrogen, 2 min in 37°C water bath, four times repeated). Afterwards, 700 μl of Z-buffer with 1 mM DTT and 160 μl of the substrate ONPG (4 mg/ml of o-Nitrophenyl-β-D-galactopyranosid in Z-buffer) were added and incubated at 30°C until colour changes into yellow. The reaction was stopped by the addition of 1 M Na_2_CO_3_ and the OD_420nm_ was measured with a spectrophotometer (UV-1601 from Shimadzu). The relative readthrough activity was calculated from the ratio of luciferase to β-galactosidase activity measured with the stop codon-containing reporter related to the ratio from the in-frame control.

### Statistical analysis

**Figure 1.**
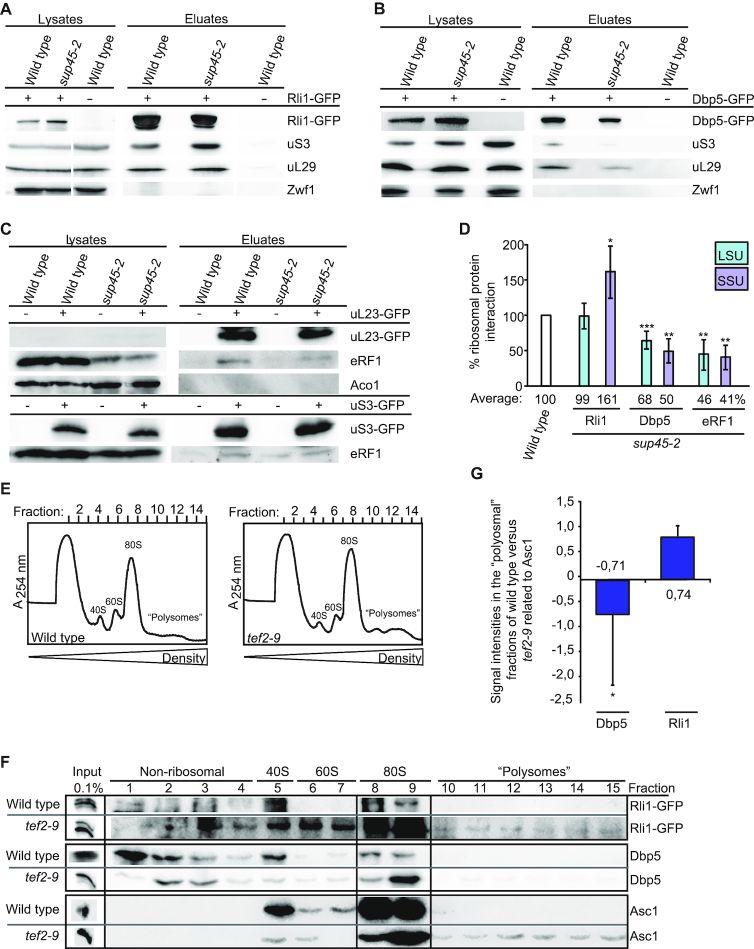
Dbp5, but not Rli1, requires eRF1 to associate with the ribosome at a stop codon. (**A**) The interaction of Rli1 with the ribosome is not dependent on eRF1. Western blot analysis of Rli1 co-IPs in wild-type and *sup45-2* cells shifted to 37°C for 1 h reveal co-precipitation of the small ribosomal protein uS3 ( = Rps3) and the large ribosomal protein uL29 ( = Rpl35). Detection of Zwf1 served as non-binding control. (**B**) The interaction of Dbp5 with ribosomal subunits is decreased in *sup45-2*. Western blot analysis shows co-precipitation of uS3 and uL29 with Dbp5 IPs in wild-type and *sup45-2* cells, shifted to 37°C for 1 h. (**C**) The interaction of mutant eRF1 with the ribosome is decreased. Western blot analysis of the co-precipitation of mutant eRF1 (sup45-2) with uL23 (top) or with uS3 (bottom) is shown. Aco1 served as a negative control. (**D**) Quantification of at least three independent experiments, one of which is shown in panels (A–C), which determine the amount of the co-precipitated proteins, measured with the Fusion SL detection system. (**E**) Ribosome profiles of wild-type and the translation elongation defective strain *tef2-9* reflects the translational run-off in wild-type and a translational arrest in the elongation mutant. Wild-type and *tef2-9* cells were shifted to 37°C for 1 h before the lysates were analysed in linear sucrose-density gradients without cycloheximide. (**F**) Rli1, but not Dbp5, is bound to ribosomes during translation elongation. Western blot analysis of the fractions, representing the total cellular amount of the indicated proteins, reveals their ratio in the 80S, polysomal or non-ribosomal, 40S and 60S fractions. Asc1 as a ribosome binding protein served as a positive control. (**G**) Quantification of four different western blot analyses shown in panel (F); *P < 0.05; **P < 0.01; ***P < 0.001.

**Figure 2.**
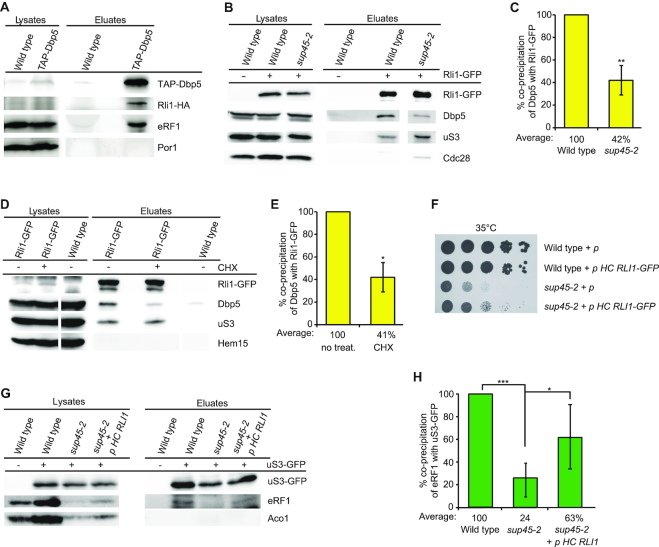
Rli1 supports the recruitment of Dbp5 and eRF1 to the ribosome. (**A**) Dbp5 interacts RNA-independently with Rli1. Immunoprecipitation of TAP-Dbp5 in the presence of RNase A shows co-precipitation of Rli1-HA in western blot analysis. Detection of eRF1 served as positive and of Por1 as negative control. (**B**) The interaction between Rli1 and Dbp5 is decreased in *sup45-2*, shifted to 37°C for 1 h. Western blot analyses of Rli1-IPs reveal less co-precipitation of Dbp5, but no reduction of the ribosomal protein uS3 in *sup45-2* compared to wild-type. Cdc28 served as negative control. (**C**) Quantification of four different experiments shown in panel (B). (**D**) Inhibition of translation elongation leads to a reduced interaction between Rli1 and Dbp5. Western blot analyses of co-IPs of Dbp5 with Rli1 upon treatment with 0.5 mg/ml cycloheximide (CHX) for 30 min are shown. Hem15 was detected as non-binding control. (**E**) Quantification of three different experiments shown in panel (D). (**F**) Overexpression of *RLI1* partially rescues the growth defects of *sup45-*2, while the wild-type growth is not influenced. Serial dilutions of the indicated strains are shown upon growth on selective plates for 3 days at 35°C. (**G**) Overexpression of *RLI1* suppresses the binding defect of eRF1 to the ribosome in *sup45-2* cells. Co-IPs of eRF1 with uS3-GFP are shown in the indicated strains with or without high copy (HC) *RLI1*. (**H**) Quantification of four different experiments shown in panel (G); *P < 0.05; **P < 0.01; ***P < 0.001.

**Figure 3.**
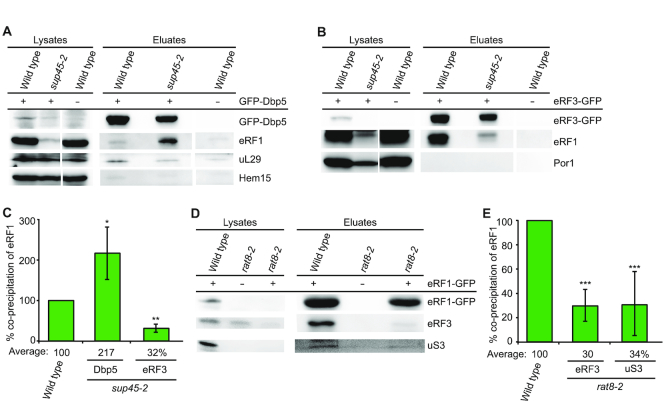
Not eRF1 and eRF3 enter the ribosome together, but eRF1 and Dbp5. (**A**) The binding of Dbp5 with the ribosome-binding defective protein sup45-2 is increased, while its ribosome association is decreased. Western blot analyses of co-IPs with mutated eRF1 (sup45-2) and Dbp5 or uL29 are shown. Detection of Hem15 served as a non-binding control. (**B**) The interaction between eRF3 and eRF1 is decreased in the *sup45-2* strain as shown in western blots of the eRF1 co-IP with eRF3. Por1 served as negative control. (**C**) Quantification of three different experiments shown in panels (A) and (B). (**D**) The interaction of eRF1 and eRF3 is decreased in a *DBP5* mutant. Western blot analysis of eRF3 co-IPs with eRF1 in wild-type and the *rat8-2* stain is shown. (**E**) Quantification of three different experiments shown in (D); *P < 0.05; **P < 0.01; ***P < 0.001.

**Figure 4.**
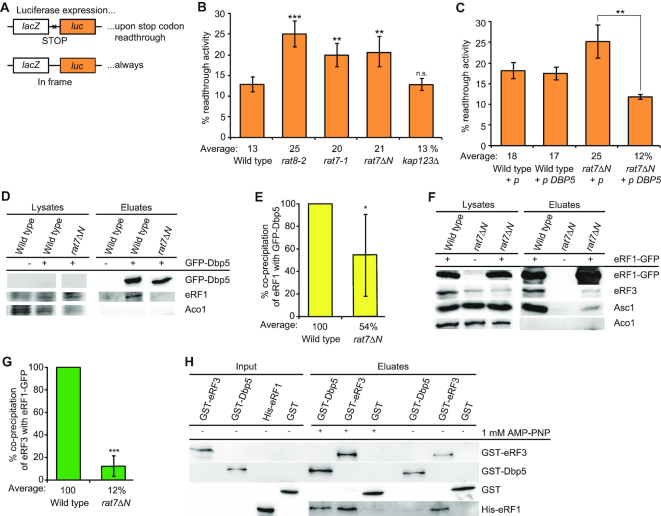
Nup159 recycles Dbp5-ATP also for translation termination. (**A**) Scheme of the reporter plasmids used in the dual reporter β-galactosidase luciferase assay. The *lacZ* gene, expressing β-galactosidase and the *luc* gene, expressing luciferase is either separated by the stop codon UAG or in frame. In the upper case, luciferase will only be expressed in case the stop codon is readthrough. The in-frame reporter serves as control to monitor basal expression levels and relate it to the stop codon containing construct. (**B**) Mutants of *NUP159* show increased readthrough of the stop codon. The average readthrough activity of at least three independent experiments is shown after shift of all indicated strains to 37°C for 30 min. (**C**) High copy *DBP5* rescues the increased stop codon readthrough of *rat7ΔN*. All strains were shifted to 37°C for 30 min. (**D**) The interaction of Dbp5 and eRF1 is disturbed in the recycling defective mutant *rat7ΔN*. Western blot analysis of a co-IP with Dbp5 and eRF1 is shown. Aco1 served as a negative control. (**E**) Quantification of three different experiments shown in panel (D). (**F**) The interaction of eRF1 and eRF3 and the ribosome is diminished in *rat7ΔN* cells. Western blot analysis of a co-IP with eRF1-GFP and eRF3 or the ribosome bound protein Asc1 is shown. (**G**) Quantification of three different experiments shown in panel (F). (**H**) Dbp5 and eRF1 directly interact in the presence of a non-hydrolysable ATP-analogue. An *in vitro* binding study with recombinant proteins in which GST-tagged Dbp5 or eRF3 were used in pull-down experiments in the presence of His-eRF1 and if indicated 1 mM AMP–PNP is shown in western blot analysis. GST alone served as a non-binding control; *P < 0.05; **P < 0.01; ***P < 0.001.

Quantification was performed for at least three independent experiments. Quantification of co-immunoprecipitation experiments shown in Figures 1D, 2C, E, 3C, E, 4B, C, E, G6D, G and I were analysed for significance by Student’s two-tailed, two-sample, unequal variance *t*-test. In all cases, each dataset was normalized and only compared to wild-type. The mean ± standard deviations are displayed. The same was done for Figures 1G, 2H and 6B, with the exception that the differences were analysed by a Student’s one-tailed, two-sample, unequal variance *t*-test. In Figures 1G and 6B, the signal intensity of the analysed proteins in the polysomal fraction of the *tef2-9* strain was subtracted from the wild-type signals in the polysoms of the respective protein and normalized to the Asc1/uS3 protein content that reflected the amount of polysomes. All data were finally analysed for significance by Student’s two tailed, two-sample, unequal variance *t*-test. Significance *P*-values below 0.05 were indicated by asterisks (*P < 0.05; **P < 0.01; ***P < 0.001).

## RESULTS

### Binding of Dbp5, but not Rli1, to the termination complex depends on eRF1

Previous studies of the translation termination process have not revealed, how and when Rli1 and Dbp5 are recruited to the terminating ribosome. Crystal structure and *in vitro* analyses with purified proteins proposed that Rli1 and eRF3 bind to the same position on the terminating ribosome so that their association seemed to be mutually exclusive and it was suggested that Rli1 might take over the binding site of eRF3 after its dissociation to catalyse the subsequent ribosome recycling (4,29) (Supplementary Figure S1B). However, a potentially earlier association of Rli1 with the ribosome before ribosome recycling has not been investigated. The same is unclear for Dbp5. The helicase might already be associated with elongating ribosomes or it could be recruited at a later step together with eRF1 upon arrival of the ribosome at the stop codon. To address these questions, we analysed the ribosomal association of Rli1 and Dbp5 in the temperature-sensitive eRF1 mutant *sup45-2* that is defective in translation termination and in ribosome binding. Upon a temperature shift to 37°C, ribosome binding of the mutated eRF1 protein sup45-2 is disturbed, resulting in stop codon recognition defects and subsequent readthrough activity in which near-cognate tRNAs are incorporated at the termination codon and translation elongation continues to the next stop codon (30). If Rli1 would enter the termination process after eRF1 has entered, one would expect to see a reduced binding of Rli1 to ribosomes in that mutant. However, co-immunoprecipitation experiments (co-IPs) show that the binding of Rli1 to the large ribosomal protein uL29 (yeast Rpl35) is unchanged and its association with the small ribosomal protein uS3 (yeast Rps3) is even increased in *sup45-2* cells (Figure 1A and D). These results indicate that Rli1 binds ribosomal particles without functional eRF1 and that it is possibly associated with ribosomes before eRF1 enters. The increased binding of Rli1 to the 40S subunit in *sup45-2* might represent an enhanced presence of 43S pre-initiation complexes, which are stabilized by Rli1 (22). In contrast to Rli1, the interaction of Dbp5 and the mutated eRF1 protein with both ribosomal proteins is reduced in *sup45-2* (Figure 1B–D) suggesting that Dbp5 requires functional eRF1 for its association with terminating ribosomes. This reduced ribosomal association might be due to the fact that mutant eRF1 is rather unstable and only ∼50% of the protein amount is detectable in lysates on western blots (Figure 1C). Thus, while the association of Rli1 with the ribosome rather increases in *sup45-2* cells, the association of Dbp5 and mutant eRF1 decreases. These results might indicate that Rli1 associates with the ribosome before Dbp5 and eRF1.

To verify this sequential recruitment, we analysed whether Rli1, but not Dbp5 associates with ribosomes arrested in translation elongation and found that this is indeed the case (Figure 1E–G). Mutations in eEF1A (such as in *tef2-9*) lead to defects in translation elongation (31), which is reflected in polysomal profiles of sucrose-density gradient fractionations that were prepared without the usual addition of cycloheximide. Under such conditions, wild-type cells continue elongation, leading to a complete polysome run-off, while elongation factor mutants stall ribosomes during elongation on the mRNA, thereby preventing their arrival at the stop codon (Figure 1E) (32). Western blot analyses of the corresponding protein fractions show that high amounts of Rli1 are present in the mono- and polysomal factions in *tef2-9* cells, similar to the ribosome-bound protein Asc1 (Figure 1G). This finding is in agreement with structural analyses of the human homolog of Rli1, ABCE1 at the ribosome, which show that the protein binds to the intersubunit space of the ribosome where aEF1 also associates (29), suggesting that their binding is mutually exclusive. However, as stalled ribosomes have free A-sites when the elongation factor is inactivated as in the *tef2-9* mutant, different factors can stochastically go there, among them Rli1. These findings suggest that Rli1 can bind to the ribosome as soon as the A-site is free and thus might be the first termination factor that enters the ribosome, which is clearly earlier than anticipated. In contrast to that, Dbp5 is almost absent in the polysome fractions of *tef2-9* cells (Figure 1F and G), indicating that Dbp5 is recruited to ribosomes only after translation elongation. Thus, our results suggest that the ribosomal association of Dbp5 not only requires a free A-site like this is the case for Rli1, because in contrast to Rli1 Dbp5 is not associated with ribosomes in an elongation mutant, but Dbp5 recruitment also seems to depend on a stop codon and functional eRF1.

### Dbp5 and Rli1 interact with each other during translation termination

Although both Rli1 and Dbp5 were identified as translation termination factors (10,13), it is unclear whether they interact with each other. To answer that question, we co-precipitated Rli1 with Dbp5 *in vivo* (Figure 2A) and vice versa Dbp5 as well as its co-factor Gle1 with Rli1 (Supplementary Figure S1B) unrevealing a physical interaction between Dbp5 and Rli1 that might be direct or mediated by other proteins. To verify that this interaction actually occurs during translation termination and not for instance during pre-ribosomal subunit export from the nucleus in which Rli1 and Dbp5 are both involved (18,19,21), we compared their interaction in *sup45-2* cells in which the termination process is inhibited and Dbp5 does not enter the ribosome. Our results show that in *sup45-2* the interaction of Rli1 and Dbp5 is indeed reduced to more than half, while its interaction to the ribosome is not decreased (Figure 2B and C). Moreover, we treated wild-type cells with the antibiotic cycloheximide, which inhibits translation elongation and thus, prevents ribosomes from arriving at stop codons, reflected in the ribosomal profiles. Also in this case the prevention of translation termination leads to a significantly decreased association of Rli1 and Dbp5 (Figure 2D and E). These results suggest that an interaction of Dbp5 and Rli1 takes place during translation termination and that both proteins bind simultaneously to the termination complex, possibly during stop codon recognition. This finding contradicts older models, in which Rli1 binds only for ribosome recycling.

An *in vivo* interaction of Dbp5 and eRF1 was shown earlier (10). However, as Rli1 most likely binds to the ribosome before Dbp5 and eRF1 have entered (Figure 1), we wondered whether Rli1 might support their recruitment to the ribosome. We therefore investigated if an overexpression of *RLI1* would suppresses the *sup45-2* mutant, which has defects in ribosome binding (Figure 1C and D), (30). Indeed, growth analyses show at least a partial rescue of the *sup45-2* growth defects in the presence of high copy *RLI1* (Figure 2F). The reason for this suppression might be that an increased amount of Rli1 proteins leads to their faster ribosome binding, which supports the defective sup45 protein in associating with the ribosome. This seems indeed to be the case as shown by co-IPs (Figure 2G). While the association of the mutated eRF1 protein with the ribosome is reduced to a quarter, increased Rli1 concentrations support the binding of sup45-2 to more than 60% (Figure 2H). These findings suggest that the initial presence of Rli1 at the ribosome could support the subsequent eRF1 recruitment to the stop codon.

### eRF1 and eRF3 do not enter the termination complex together

In contrast to Rli1, Dbp5 requires intact eRF1 for its binding to the ribosome (Figure 1). From previous studies, it was suggested that Dbp5 might help to position eRF1 at the stop codon and dissociate before eRF3 enters the termination complex (10). Thus, it seems conceivable that eRF1 and eRF3 are not recruited as a complex, but rather individually, which challenges current models. To analyse whether Dbp5 and eRF1 form a complex already in the cytoplasm, we took again advantage of the *sup45-2* mutant, in which the mutated eRF1 protein sup45-2 has a ribosome-binding defect and is thus detached and freely present in the cytoplasm at the non-permissive temperature (Figure 1C and D) (30) and analysed its binding to Dbp5. Indeed, co-IPs revealed an increased binding of the cytoplasmic sup45-2 to Dbp5 (Figure 3A and C), while at the same time its interaction to eRF3 is decreased (Figure 3B and C). Remarkably, despite the fact that the sup45-2 protein is less stable as compared to wild-type eRF1, as reflected in the lysate lanes, its binding to Dbp5 is significantly increased. These results could suggest that a pre-formed complex of Dbp5 and eRF1 in the cytoplasm approaches the terminating ribosome, while eRF3 enters separately upon Dbp5-dissociation. As Dbp5 and eRF3 do not interact *in vivo* (10), a complex formation between the three termination factors is unlikely or their potential contact is very short.

To further investigate whether Dbp5 might indeed deliver eRF1 to the ribosome and eRF1 and eRF3 do not enter the ribosome together, we analysed if mutations in *DBP5* would lead to a reduced binding of eRF1 to eRF3 and to the ribosome. For this purpose, we used the *rat8-2* strain that produces instable Dbp5 protein due to a leucine to proline exchange at position 267 (33) (Figure 6H). Indeed, *in vivo* interaction studies of eRF1 and eRF3 in *rat8-2* mutants reveal a ∼70% reduction of eRF1 binding to the ribosome and to eRF3 upon a 1 h temperature shift to 37°C (Figure 3D and E). The reduced eRF1 and eRF3 interaction was also detected earlier and seems to happen immediately, already after a 20 min temperature shift of the *rat8-2* strain. However, the ribosomal binding of eRF1 was less obviously decreased after this short shifting time (10). But the longer 1 h shift produces a clear ribosome binding defect of eRF1 (Figure 3D and E). Together, our findings suggest that Dbp5 might deliver eRF1 to the ribosome without eRF3.

### Nup159 recycles Dbp5–ADP for export and translation termination

The ATPase activity of Dbp5 is essential not only for mRNA transport, but also for translation termination (10,16,17). During termination, we suggest that the helicase might deliver eRF1 and it seems possible that it could use its ATPase-dependent activity to position eRF1 properly on the stop codon. In particular, because we have shown earlier that its ATPase activity is necessary not only for its function in mRNA export, but also for its role in translation termination (10). In both cases, upon ATP-hydrolysis, the enzyme needs to be recycled. During mRNA export, the nucleoporin Nup159/Rat7 is the ADP-release factor of Dbp5 (17). Thus, it is conceivable that recycling from termination also occurs at the NPC via Nup159, rather than at the ribosome. In particular, because Dbp5 must return to the cytoplasm to capture a new molecule of eRF1 as they first interact in the soluble fraction of the cytosol (Figure 1). Indeed, readthrough experiments with a dual β-galactosidase luciferase reporter system show an increased readthrough activity in different *nup159* mutants, very similar to the *dbp5* mutant *rat8-2* (Figure 4A and B), (10). As both *nup159* mutants, *rat7-1* and *rat7ΔN*, the latter of which specifically lacks the interaction domain for Dbp5 (34), exhibit increased readthough activities as compared to wild-type (Figure 4B), Nup159 seems to be the recycling factor for Dbp5 not only for mRNA export, but also for translation termination. In support of this model, we found that overexpression of *DBP5* leads to a rescue of the high readthrough activity in *rat7ΔN* cells (Figure 4C) indicating that less recycling by Nup159 is needed when more Dbp5–ATP is present.

These results suggest that Nup159 recycles Dbp5–ADP also upon its action in translation termination, which is quite attractive, because in this way Dbp5 might couple two important cellular processes—nuclear mRNA export and translation. When the translation rate is low, Dbp5 is free to increasingly act in mRNA export to raise the mRNA amount in the cytoplasm and vice versa, high translation rates could reduce mRNA export. To verify a dependence of the eRF1–Dbp5 interaction on Nup159, because their contact should only be established when Dbp5 is ATP-bound, we performed co-IPs with these proteins in *rat7ΔN*. Indeed, while the interaction of Dbp5 and eRF1 was clearly detectable in wild-type, it was significantly reduced in *rat7ΔN* (Figure 4D and E), supporting a model in which Dbp5 is recycled at the NPC with ATP. These findings further suggest that Dbp5–ATP can bind eRF1, while Dbp5–ADP might release the termination factor. As Dbp5 could not be re-charged in *rat7ΔN* and thus cannot deliver eRF1 to the ribosome anymore, the interaction of eRF1 with eRF3 should also be decreased in this mutant, which is indeed the case as shown by co-IPs (Figure 4F and G).

The switch in eRF1 binding and release through ATP-hydrolysis of Dbp5 was further investigated in *in vitro* experiments with purified recombinantly expressed proteins. We show that Dbp5 only binds to eRF1 in the presence of the non-hydrolysable ATP-analogue AMP–PNP, whereas eRF3 interacts also ATP-independently with eRF1 (Figure 4H). Together, these *in vivo* and *in vitro* studies support a model in which eRF1 associates with Dbp5–ATP in the cytoplasm and dissociates from Dbp5–ADP at the ribosome, where Dbp5 possibly uses its ATP-dependent helicase activity to place eRF1 properly on the stop codon. Moreover it becomes evident that eRF1 and Dbp5 form a complex in the cytoplasm, from which eRF3 is absent.

### Binding of Dbp5 and eRF3 to eRF1 is mutually exclusive

**Figure 5.**
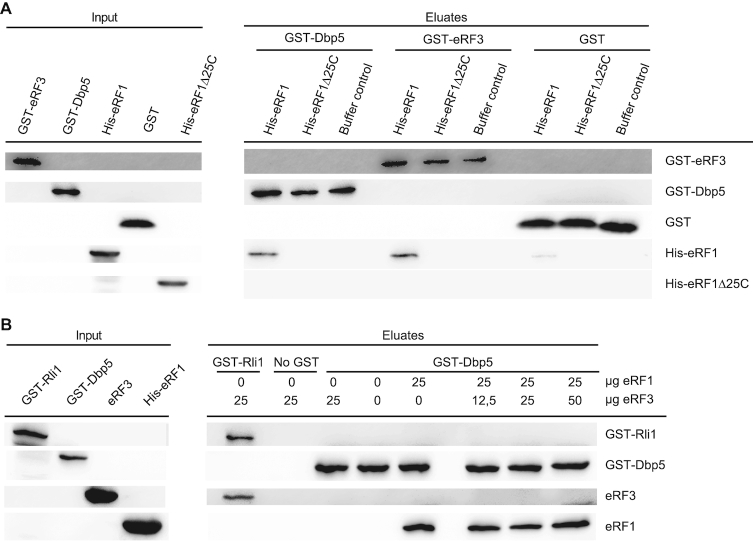
Dbp5 and eRF3 interaction with eRF1 is mutually exclusive. (**A**) The interaction site of eRF1 with Dbp5 and eRF3 overlap. Western blot analyses of pull-downs experiments with GST–Dbp5, GST–eRF3 or GST and His-eRF1 or His-eRF1∆25 lacking the last C-terminal amino acid residues are shown. All binding buffers contained 1 mM AMP–PNP. (**B**) A preformed complex of eRF1 and Dbp5 cannot be disrupted by eRF3. Western blot analysis of a competition assay of the indicated recombinantly expressed proteins is shown. Increasing amounts of eRF3 were added to the preformed complex of GST–Dbp5 and His-eRF1. Rli1 served as a positive control for eRF3 binding. All binding buffers for Figure 5 contained 1 mM AMP–PNP.

As Dbp5 and eRF1 enter termination complexes together, and Dbp5 does not interact with eRF3 (10), it seems possible that the binding of Dbp5 and eRF3 with eRF1 is mutually exclusive. It was shown that the eRF3-interaction domain of eRF1 comprises the last 25 amino acid residues of its C-terminus (5). *In vitro* binding studies with recombinant proteins were carried out to investigate whether this domain is also the Dbp5-interaction domain. Indeed, while full-length eRF1 interacts with both, eRF3 and Dbp5, no interaction was detectable with eRF1Δ25C (Figure 5A), indicating that both termination factors share the same binding site on eRF1. Interestingly, although also the middle domain of eRF1 was reported to contribute to the eRF1–eRF3 interaction (35), we found that the deletion of the C-terminal domain is sufficient to abrogate the interaction of eRF1 with eRF3 and Dbp5 *in vitro*. Moreover, a preformed interaction of Dbp5 and eRF1 was not disrupted by the addition of increasing amounts of eRF3 in a competition assay (Figure 5B). Intriguingly, these findings suggest indeed a sequential and mutually exclusive binding of Dbp5 and eRF3 to eRF1 with a first complex formation between Dbp5 and eRF1. Thus, a model is possible, in which during the progress of termination, Dbp5–ATP prohibits the access of eRF3 to eRF1 until eRF1 was placed properly in the ribosomal A-site. Such a mechanism would prevent a premature access of eRF3 and a consequent premature GTP-hydrolysis. Because as soon as eRF3 contacts its guanine exchange factor eRF1 at the ribosome, eRF3 binds GTP, which is subsequently hydrolysed, resulting in the immediate dissociation of eRF3 from the ribosome (36,37). The suggested sequential entry of the termination factors would have the advantage that the contact of eRF3 with eRF1 is controlled, which will prevent premature GTP-hydrolysis of eRF3 and its subsequent premature dissociation before the stop codon is successfully recognized.

### eRF3 binds to the ribosome prior to eRF1

**Figure 6.**
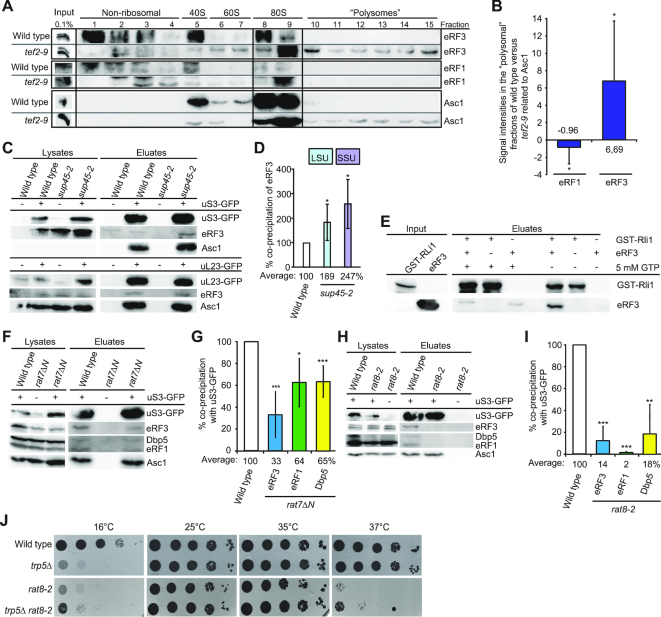
eRF3–GDP binds Rli1 prior to the entry of Dbp5 and eRF1. (**A**) eRF3 is present on ribosomes stalled in translation elongation. Western blot analysis of the collected fractions of the *tef2-9* gradient shown in Figure 1E with antibodies against eRF3, eRF1 and Asc1 are displayed. (**B**) Quantification of four different western blot analyses shown in panel (A). (**C**) Ribosome binding of eRF3 in *sup45-2* is increased. Western blot analysis of co-IPs of eRF3 and the positive control Asc1 with uS3 (top) and uL29 (bottom) are shown. (**D**) Quantification of four different IPs shown in (C). (**E**) Rli1 binds nucleotide free eRF3 directly and releases eRF3-GTP. Western blot analysis of *in vitro* pull-down experiments with Rli1 is shown. (**F**–**I**) The ribosomal association of eRF1, eRF3 and Dbp5 is decreased in *nup159* (F and G) or *dbp5* (H and I) mutants. Western blot analyses of the uS3-co-precipitated proteins in the indicated strains are shown. (**G** and I) Quantification of four (G) and three (I) different IPs shown in panels (F) and (H), respectively. (**J**) Defects in eRF1 delivery partially suppresses the growth defects of *trp5Δ*. Serial dilutions of the indicated strains are shown upon growth on full medium agar plates at the indicated temperatures; *P < 0.05; **P < 0.01; ***P < 0.001.

Protection of eRF1 from premature eRF3 access would only be necessary if eRF3 would already be present at the ribosome when eRF1 enters. Therefore, we analysed its ribosomal association in the *tef2-9* mutant that arrests in translation elongation as shown in Figure 1E and F. Strikingly, eRF3, but almost no eRF1 is detectable in the polysomal fractions of this elongation mutant, suggesting that eRF3 can independently bind ribosomes before they arrive at a stop codon and is therefore already present when eRF1 enters (Figure 6A and B). These findings are supported by co-immunoprecipitation analyses of eRF3 with ribosomal proteins in the mutant *sup45-2*. In the situation in which this mutant eRF1 protein accumulates with Dbp5 in the cytoplasm (Figure 1), the binding of eRF3 to the ribosomal protein uS3 is not reduced, but rather increases as its eRF1 mediated GTP-hydrolysis and release is prevented (Figure 6C and D).

Because Rli1 is also present at that early time point, we investigated a potential direct interaction of eRF3 and Rli1. *In vitro* binding studies revealed that either nucleotide free eRF3, or GDP-bound but not GTP-bound eRF3 directly interacts with Rli1 (Figure 6E), (23), suggesting that Rli1 might bind to eRF3–GDP at the ribosome, where they wait for the arrival of eRF1. Furthermore, Rli1 interacts with eRF3 without the addition of ATP (Figure 6E), supporting a model in which nucleotide-free Rli1 binds to the ribosome and eRF3 and the recruitment of ATP to Rli1 occurs later. As this nucleotide-free state of Rli1 was shown to result in a rather weak association with the ribosome (23), it is conceivable that Rli1 might change its position on the ribosome during the stepwise assembly of all termination factors. Upon eRF1 entry, eRF3 most likely binds to GTP, as its affinity to the triphosphate increases upon eRF1 contact (36,37), which would trigger its dissociation from Rli1, because it interacts only with eRF3–GDP (Figure 6E). These rearrangements in the termination complex through eRF3–GTP-hydrolysis stimulate the eRF1-mediated polypeptide- and tRNA-release by moving eRF1 into its favourable position to terminate translation (37). The poly(A) binding protein Pab1 might further support this early association of eRF3 to the ribosome and Rli1 as eRF3 and Pab1 were shown to interact (38,39).

### Dbp5 enables a stable contact of eRF1 and eRF3 at the ribosome

In a model in which Dbp5 delivers eRF1 and prevents premature excess of eRF3, one would expect that in a situation in which the Dbp5 binding to eRF1 is inhibited, the release factor might be able to access the ribosome alone and immediately contact eRF3. This situation should result in the immediate dissociation of eRF1 and eRF3 from each other and from the ribosome, because their contact would not be prevented until eRF1 was properly positioned by Dbp5. This is indeed the case. In a mutant of the Dbp5 recycling factor Nup159, *rat7ΔN*, Dbp5 remains ADP-bound, which prevents its complex formation with eRF1 (Figure 4D), or in mutant *DBP5*, such as *rat8-2*, in which the protein is detached from the NPC at 37°C and not re-charged with ATP (40), eRF1 is not Dbp5 bound. In both cases, the freely available eRF1 leads to the reduced presence of eRF1 and eRF3 at the ribosome as reflected in uS3 co-IPs (Figure 6F–I). Possibly eRF1 and eRF3 are less present at the ribosome, because both release factors instantly dissociate upon their uncontrolled contact, because their contact initiates the GTP-binding of eRF3, its subsequent hydrolysis, which triggers the dissociation of eRF1 and eRF3. Such premature contact would support the observed increased readthrough activity (Figure 4B and C).

It is quite intriguing that Dbp5 controls the entry of eRF1 into termination reactions. However, this novel function might have an impact not only at termination codons on readthrough activity, but also on termination events at near cognate codons, such as the UGG tryptophan codon that is similar to the stop codon UGA. In such situation it is observed that mRNA translation is not continued and instead translation termination occurs. To investigate whether Dbp5 would genetically interact with the tryptophan synthetase mutant *trp5Δ*, we generated the *rat8-2 trp5Δ* double mutant and monitored the growth at different temperatures. We found that the double mutant partially suppresses both single mutants at 16°C and *rat8-2* strain also at higher temperatures. This suppression phenotype suggests that mutations in *DBP5* indeed affect termination events at near cognate codons and the deleterious effect of the trp5 deletion stems from inefficient decoding of UGG codons, and increased eRF1-catalysed mistermination events on such codons. This effect appears suppressed in *rat8-2* mutants where the delivery of eRF1 to such codons would be reduced (Figure 6J).

### eIF3 enters translation termination after the Dbp5-mediated delivery of eRF1

**Figure 7.**
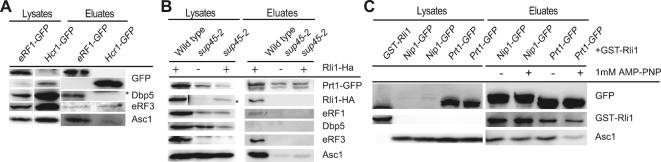
The eIF3 complex binds to the ribosome after the Dbp5-mediated delivery of eRF1. (**A**) The eRF3 release factor Hcr1 does not bind to Dbp5 and only weakly to the ribosome. Co-IPs with GFP-tagged Hcr1 and as a control eRF1–GFP with Dbp5, eRF3 and the ribosomal protein Asc1 are shown. The asterisk indicates Hcr1–GFP. (**B**) Defects in the eRF1 delivery result in the absence of the eIF3 subunit Prt1 at the ribosome. Co-IPs of GFP-tagged Prt1 with HA-tagged Rli1, eRF1, Dbp5, eRF3 and the ribosomal protein Asc1 are shown in wild-type and *sup45-2* strains. The asterisk indicates that the *sup45-2* lysate lanes were exposed four times as long as the wild-type lanes. (**C**) The binding of the eIF3 subunits Prt1 and Nip1 are independent of the nucleotide association of Rli1. The GFP-tagged eIF3 subunits were precipitated and the co-precipitated GST-tagged Rli1 and Asc1 are shown.

In addition to eRF1, eRF3, Rli1 and Dbp5, the translation initiation factor, eIF3 with its subunit Hcr1 were shown to participate in translation termination (15). Mutations in eIF3 reduce the rate of stop codon readthrough, while *hcr1Δ* shows an increased readthrough activity and this phenotype was suppressed by high copy Rli1. A model was proposed in which Hcr1 is not a bona fide translation initiation factor, but rather acts in termination by promoting GDP–eRF3 ejection from the ribosomes (15). In such a model Dbp5 would not bind to Hcr1, because it would dissociate before Hcr1 would contact eRF3. To investigate this, we carried out co-IPs with Hcr1 and Dbp5 and could confirm the Hcr1 eRF3 interaction, while no interaction between Hcr1 and Dbp5 was visible (Figure 7A). Interestingly, also the interaction of Hcr1 with the ribosome is less strong as that of eRF1, supporting the model that Hcr1 ejects eRF3 from the ribosome. eIF3 was suggested to promote ribosome recycling after termination. As such, one would expect the complex to bind after translation termination. Indeed, one of the eIF3 subunits Prt1 clearly interacts with Rli1, eRF3, eRF1 and the ribosome, represented by Asc1 in wild-type cells, but these interactions were abrogated in *sup45-2* cells, in which the termination reaction does not occur (Figure 7B). A Dbp5 Prt1 interaction was not detected, which might suggest that a potential interaction is either rather short or Prt1 associates only after Dbp5 has delivered eRF1. We further show in co-IPs that the interaction of eIF3 with Rli1 and the ribosome is independent on the ATP-binding of Rli1, because both eIF3 subunits, Prt1 and Nip1 interact without or with the addition of AMP–PNP (Figure 7C).

**Figure 8.**
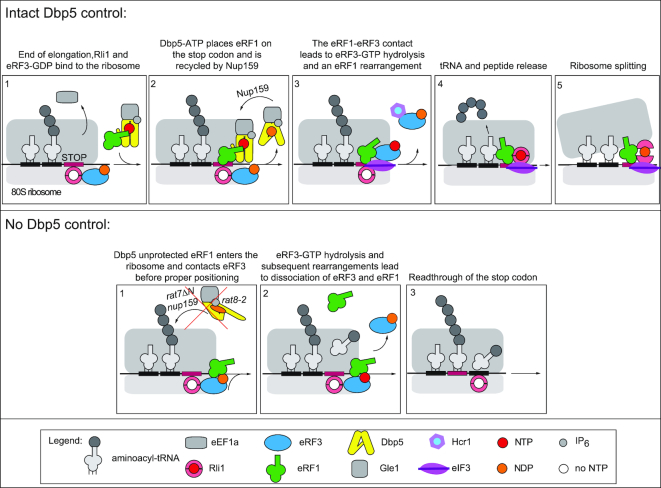
Stepwise entry model for translation termination. (Top) Step 1: Nucleotide-free Rli1 associates with the ribosomes as soon as the A-site is free. It binds to eRF3–GDP. Step 2: Rli1 supports the entry of Dbp5–ATP bound eRF1. Gle1/IP_6_ stimulated ATP-hydrolysis of Dbp5–ATP leads to the proper positioning of eRF1 on the stop codon. Dbp5–ADP dissociates and is recycled at the nuclear pore complex by Nup159. Step 3: Dissociation of Dbp5–ADP allows the controlled interaction of eRF1 with eRF3. This in turn triggers the GTP recruitment of eRF3. Subsequent GTP hydrolysis leads to conformational changes in eRF1 allowing adjustments in its positioning in the ribosomal peptidyl-transferase center. eRF3–GDP dissociates in a complex with Hcr1, which was delivered by eIF3. Step 4: eRF3–GDP dissociation allows change of position and strong binding of Rli1–ATP that locks eRF1 in the position necessary to mediate peptidyl-tRNA hydrolysis. Step 5: Upon peptide release, ATP-hydrolysis of Rli1–ATP recycles the ribosomal subunits, which is supported by eRF1. (Bottom) Situation in which Dbp5 cannot deliver eRF1 to the ribosome that consequently results in the stop codon readthrough. Step1: Rli1 associates and binds eRF3–GDP. Step 2: eRF1 is not protected by Dbp5 and contacts eRF3 before being properly positioned, leading to premature GTP-binding, hydrolysis and the subsequent release of eRF1 and eRF3–GDP from the ribosome before the polypeptide chain and the tRNA are released. Because eRF1 had contact to eRF3 before it was placed in the optimal position, it dissociates at the same time as eRF3. Step3: A near-cognate tRNA gets access to the A-site, the stop codon is suppressed and translation elongation continues until the next stop codon is reached.

These data support the following stepwise entry model for translation termination in yeast (Figure 8). Nucleotide-free Rli1 binds to the ribosome as soon as the A-site is unoccupied. eRF3–GDP associates with Rli1 and waits for the entrance of eRF1. Rli1 furthermore supports the entry of eRF1 into the ribosome, which is delivered and positioned at the stop codon by the helicase Dbp5. The contact of eRF3 and eRF1 is controlled by the dissociation of Dbp5, which occurs upon hydrolysis of its ATP through Gle1- and IP_6_-stimulation. ADP–Dbp5 recycling is mediated at the NPC through Nup159, which couples translation to mRNA-export. Dissociation of Dbp5 allows contact of eRF1 and eRF3, which stimulates eRF3 to associate with GTP and induces a stronger binding to Rli1, which might indicate that Rli1 changes its position. The subsequent GTP-hydrolysis of eRF3 results in the final positioning of eRF1, dissociation of eRF3–GDP by Hcr1, which is delivered by eIF3 to the ribosome and to peptidyl-tRNA hydrolysis and polypeptide chain release from the ribosome. Subsequent recycling of the ribosomal subunits is mediated by Rli1 (4,23,26).

## DISCUSSION

Translation termination depends on the two key factors eRF1 and eRF3, but also on Dbp5 and Rli1 (10,13). However, their function and the order in which the termination complex assembles, was unclear. Current models suggest that eRF1 and eRF3 enter the ribosome together as a complex (1,2,4), but we show here that this is very unlikely and propose a new translation termination model in which the termination complex assembles stepwise (Figure 8).

Polysomal gradients and co-IPs indicate that nucleotide-free Rli1 and eRF3–GDP binds to the ribosome prior to eRF1 and Dbp5 entry (Figures 1 and 6). Our data support a function of Rli1 in promoting the recruitment of the other termination factors. In particular, it promotes the binding of the Dbp5–eRF1 complex leading to the formation of a ternary complex with Rli1 (Figures 2F–H and 6E). For such a function, Rli1 would not require its ATPase activity, which is in agreement with earlier studies in which it was shown that ATP-hydrolysis by Rli1 does not take place during the termination process (4,13). It was furthermore suggested earlier that Rli1 associates with the termination complex upon dissociation of eRF3–GDP, taking over its position to lock eRF1 in its favourable position to facilitate peptidyl-tRNA hydrolysis (4,26). In contrast to this model, we present evidence that Rli1 and eRF3–GDP are the initial components of the termination complex that bind to the ribosome. However, our data are in agreement with a model in which in the course of the stepwise assembly, Rli1 could take over the position of eRF3, because initially eRF3 binds to Rli1 in its GDP bound form and dissociates upon GTP binding and hydrolysis (Figure 6E), which could allow the remodelling of the complex and enable Rli1 to occupy the eRF3 position upon its release. Because Rli1 is most likely nucleotide-free in this early stage of translation termination (Figure 6E), it is conceivable that the rearrangements upon eRF3 dissociation might be supported by its binding to ATP, which is later on required for the splitting of the ribosomal subunits (23,24). However, it is also possible that the entry of eIF3 and/or the dissociation of eRF3 through Hcr1 (Figure 7A–C) induce these rearrangements.

In contrast to earlier models, our results furthermore indicate that Dbp5–ATP captures eRF1 in the cytoplasm (Figures 3A,C and 4D) and delivers it to the stop codon-bound ribosome on which eRF3–GDP and Rli1 are already present (Figures 1F,G and 6A,B). The interaction between Rli1 and Dbp5 might thereby support the Dbp5–eRF1 recruitment (Figure 2). Upon placing eRF1 properly on the stop codon, Dbp5–ADP dissociates and is recycled at the NPC via Nup159 (Figure 4). In this way, the two key processes of mRNA export and translation are coupled via Dbp5. How the duty of Dbp5 in both processes is divided and how Dbp5 captures eRF1 is currently unclear and needs further investigation. Interestingly, the overall cellular protein abundance estimates suggest an intracellular Dbp5:eRF1:eRF3 ratio of ∼1:2:4 (https://www.yeastgenome.org/). Dbp5 as a limiting factor further qualifies itself as a regulator of the interaction between eRF1 and eRF3, because only the Dbp5-delivered eRF1 can engage in proper termination events. Excess of eRF1 over Dbp5 might be required, because it participates also in ribosome recycling (4,23,26) and thus remains bound for a longer time. The high amounts of eRF3 might be required, because it waits on every ribosome at late stages of elongation (Figure 6A and B) for eRF1 to enter. In support of this view, it is interesting to note that a 20% decrease of the basal cellular protein levels of Dbp5 or eRF1 already negatively impact protein biosynthesis, while a 60% decrease of eRF3 has no effect (14). Thus, the Dbp5-mediated delivery of eRF1 is the rate-limiting step, supporting a view that Dbp5 controls the termination event.

Interestingly, our *in vivo* results clearly show that Dbp5 is not bound to the ribosome during elongation (Figures 1F, G, 2D and E), although its human homolog DDX19 has been shown to stabilize translation elongation *in vitro* (14). This might either be a difference between the human and the yeast helicase, but it is also conceivable that in the *in vivo* situation a contact of Dbp5 with the elongating ribosome is prevented and its recruitment is only possible when bound to eRF1.

Importantly, the simultaneous entry of Dbp5 and eRF1 in a complex has the advantage that Dbp5 protects eRF1 from premature access to eRF3, because Dbp5 occupies the binding domain of eRF3, located at the last 25 amino acid residues of the C-terminal domain (Figure 5). This is particularly important, because eRF3 is already present at the ribosome, when Dbp5–eRF1 enters (Figure 6A and B). In fact, a separated entry of eRF1 and eRF3 is actually supported by earlier studies, showing that a reduced eRF3 expression does not lead to a decreased ribosomal association of eRF1 (3).

In situations of defective Dbp5 or Dbp5–ATP recycling, as in the *rat8-2* and *nup159ΔN* mutants, respectively, the protected eRF1 delivery to the ribosome cannot occur and eRF1 can contact eRF3 prior to its proper positioning and stop codon recognition. This contact would induce eRF3 to release GDP and bind GTP, because the affinity of eRF3 to GTP strongly increases upon eRF1 contact (36,37). This in turn can trigger the premature GTP-hydrolysis and subsequent dissociation of eRF3–GDP and eRF1 from each other and the ribosome (Figure 6F–I), as evident in these mutants (Figures 3D, E, 4F and G). In support of such a model, it is interesting to note that a reduced binding of eRF3 to polysomes was already detected in *rat8-2* cells upon a very short, 20 min temperature shift to the non-permissive temperature and in mutants of the co-factor of Dbp5, *GLE1* (10,12). As eRF1 would not have been properly positioned when Dbp5 cannot deliver it, stop codon recognition, polypeptide chain- and tRNA-release would be unsuccessful and translation might be continued with near-cognate tRNAs, resulting in the observed readthrough activity in *nup159* and *dbp5* mutants (Figure 4B and C). Longer temperature shifts of both mutants and thus constant defects in stop codon recognition and mRNA transport cannot be tolerated and are lethal to cells (33,34).

Dbp5 not only impacts stop codon readthrough, but also controls the delivery of eRF1 to near-cognate codons, such as the UGG tryptophan codon, suggested by its suppression of the *trp5Δ* strain (Figure 6J). This supports a view, in which Dbp5 controls the delivery of eRF1. This discovery further suggests that cells may use this system to trigger stop codon readthrough in particular situations. Stress for example changes the expression program of cells, as it blocks bulk mRNA export, while it allows the uncontrolled export of stress specific mRNAs (41,42). Such massive changes in the cellular expression program might also involve Dbp5. Intriguingly, it was reported that Dbp5 mislocalizes to the nucleus upon ethanol stress (43), which would circumvent the helicase to support efficient termination and rather promote the readthrough of stop codons. Also during glucose starvation, in which the ATP-production is reduced, Dbp5 is most likely not efficiently re-charged with ATP, which in turn should reduce the Dbp5-mediated eRF1 delivery and result in an increased readthrough of stop codons.

Generally, stop codon readthrough has the potential to create proteins with new or additional functions. Extended C-termini could for instance add nuclear localization signals to normally cytoplasmic proteins and in this way re-direct them to the nucleus. It is also possible that the longer protein is unstable and quickly degraded. Moreover, when no additional stop codon is present in an mRNA upon readthrough, the ribosome subsequently decodes the mRNA into the poly(A) tail. In such cases, the consequence is the degradation of the protein and the mRNA by the no-stop decay (NSD) system (44). In higher eukaryotes e.g. in Drosophila or mammals, stop codon readthrough occurs also during developmental processes and is called functional translational readthrough (FTR) (45,46). In these cases, the stop codons are suppressed and treated as sense codons due to the competition between eRF1 and near-cognate tRNAs at the A-site. It is tempting to speculate that Dbp5/DDX19 might be involved in regulating such processes.

Most interestingly, one-third of all inherited disorders are caused by protein truncating pre-termination mutations that lead to non-functional proteins or cause dominant negative effects, leading to cancer and neurodegenerative diseases (47,48). Nonsense suppression therapies comprise approaches aiming at suppressing translation termination at in-frame premature stop codons to restore the deficient protein function. Using Dbp5/DDX19 as a drug target to decrease its function and increase readthrough at premature stop codons for suppression therapy might be a novel starting point for therapies.

## Supplementary Material

Supplementary Data

## Notes

Present address: Simon Uhse, Gregor Mendel Institute of Molecular Plant Biology, Austrian Academy of Sciences, Vienna Biocenter Campus (VBC), Vienna, Austria.
