# Supplementary material for: Translation termination depends on the sequential ribosomal entry of eRF1 and eRF3
Source: Nucleic Acids Res. 2019 Mar 15;47(9):4798–813. doi: 10.1093/nar/gkz177 (PMC6511868; doi:10.1093/nar/gkz177)
Supplement: Supplementary Data [file gkz177_supplemental_file.pdf]

SUPPLEMENTARY DATA

Table S1

Saccharomyces cerevisiae strains used in this study, related to Figures 1-7

Table S2

Plasmids used in this study, related to Figures 1-7

Table S3

Oligonucleotides used in this study, related to the Methods section

Figure S1. Old model for translation termination and Rli1-GFP Co-IP. (A) Current model for translation termination and open questions. Step 1: Translation elongation is followed by termination and it is unknown when termination factors associate. Step 2: The ribosome reaches a stop codon and most models suggest that eRF1 associates with the ribosome together with eRF3-GTP as ternary complex. eRF1 recognizes the stop codon. The time point of Dbp5-entry is currently unknown and whether Nup159 or another unknown cytoplasmic ADP-release factor recycles Dbp5. GTP-hydrolysis of eRF3 leads to conformational changes in eRF1 resulting in its proper positioning in the ribosomal peptidyl-transferase center. Step 3: The dissociated eRF3-GDP enables Rli1 entry and the locking of eRF1 in a position that allows peptidyl-tRNA hydrolysis. Step 4: Upon peptide release, Rli1 uses its ATPase activity to recycle eRF1 and the ribosomal subunits. (B) Rli1 physically interacts with Dbp5. Western blot analysis of a Dbp5-IP analyzing the binding of Rli1. eRF1 served as positive and Hem15 as negative control. Related to Figure 2A.

Table S1. *Saccharomyces cerevisiae* strains used in this study.

Strain number	Genotype	Source
HKY36	*MATα ura3-52 leu2Δ1 his3Δ200*	[1]
HKY124	*MATα ura3-52 leu2Δ1 his3Δ200 rat7-1*	[2]
HKY130	*MATa ura3-52 leu2Δ1 trp1Δ63 rat8-2*	[3]
HKY133	*MATα ura3-52 leu2Δ1 his3Δ200 kap123::HIS3*	[4]
HKY291	*MATα ura3-52 leu2Δ1 his3Δ200 trp1Δ63*	[5]
HKY314	*MATa ura3Δ0 leu2Δ0 his3Δ1 met15Δ0*	Euroscarf
HKY400	*MATα ura3-52 trp1-Δ lys2-20 leu2-3,112 his4-713 met2-1 tef2-Δ2 tef1::LEU2 + pCEN tef2-9 URA3*	[6]
HKY445	*MATα ura3-1 his5-2 can1-100*	[7]
HKY446	*MATα ura3-1 ade2-1 his5-2 can1-100 sup45-2*	[7]
HKY473	*MATa ura2 leu2 ade2 trp1 lys2 sup45-2*	[5]
HKY474	*MATa ura2 leu2 trp1*	This study
HKY477	*MATa ura3-52 leu2Δ1 his3Δ200 trp1Δ63*	This study
HKY522	*MATα pheA10 ade2-144 his7-1 lys9-A21 trp1-289 ura3-52 leu2-3 sup35-21*	[8]
HKY553	*MATα pheA10 ade2-144 his7-1 trp1-289 ura3-52 leu2-3*	[8]
HKY617	*MATa ade2 arg4 leu2-3,112 trp1-289 ura3-52 DBP5:TAP:K.l.URA3*	Euroscarf
HKY618	*MATa ade2 arg4 leu2-3,112 trp1-289 ura3-52*	Euroscarf
HKY1121	*MATa ura3Δ0 leu2Δ0 his3Δ1 met15Δ0 NIP1:GFP:HIS3MX6X*	Invitrogen
HKY1122	*MATa ura3Δ0 leu2Δ0 his3Δ1 met15Δ0 PRT1:GFP:HIS3MX6X*	Invitrogen
HKY1123	*MATa ura3Δ0 leu2Δ0 his3Δ1 met15Δ0 RPG1:GFP:HIS3MX6X*	Invitrogen
HKY1271	*MATa ura3Δ0 leu2Δ0 his3Δ1 met15Δ0 RLI1:GFP:HIS3MX6X*	Invitrogen
HKY1646	*MATa ura3-52 leu2Δ1 trp1Δ63 his3Δ200 rat7ΔN*(1-500)	[9]
HKY1907	*MATa ura3Δ0 leu2Δ0 his3Δ1 met15Δ0 TRP5::KANMX4*	Invitrogen
HKY1914	*MATα ura3Δ0 leu2Δ0 his3Δ1 met15Δ0 PRT1:GFP:HISMX6*	This study
HKY1915	*MATα ura3Δ0 leu2Δ0 his3Δ1 met15Δ0 PRT1:GFP:HISMX6 sup45-2*	This study
HKY1921	*MATa ura3-52 leu2Δ1 his3Δ200 trp1Δ63 DBP5::HISMX6 TRP5::KANMX4*	This study
HKY1948	*MATα ura3Δ0 leu2Δ0 his3Δ1 met15Δ0 HCR1-GFP:HISMX6*	Invitrogen

References for Table S1

1. Winston, F., C. Dollard, and S.L. Ricupero-Hovasse, *Construction of a set of convenient Saccharomyces cerevisiae strains that are isogenic to S288C.* Yeast, 1995. **11**(1): p. 53-5.
2. Del Priore, V., et al., *A structure/function analysis of Rat7p/Nup159p, an essential nucleoporin of Saccharomyces cerevisiae.* J Cell Sci, 1997. **110 (Pt 23)**: p. 2987-99.
3. Snay-Hodge, C.A., et al., *Dbp5p/Rat8p is a yeast nuclear pore-associated DEAD-box protein essential for RNA export.* EMBO J, 1998. **17**(9): p. 2663-76.
4. Seedorf, M. and P.A. Silver, *Importin/karyopherin protein family members required for mRNA export from the nucleus.* Proc Natl Acad Sci U S A, 1997. **94**(16): p. 8590-5.
5. Gross, T., et al., *The DEAD-box RNA helicase Dbp5 functions in translation termination.* Science, 2007. **315**(5812): p. 646-9.
6. Dinman, J.D. and T.G. Kinzy, *Translational misreading: mutations in translation elongation factor 1alpha differentially affect programmed ribosomal frameshifting and drug sensitivity.* RNA, 1997. **3**(8): p. 870-81.

7. Stansfield, I., et al., *A conditional-lethal translation termination defect in a sup45 mutant of the yeast Saccharomyces cerevisiae.* Eur J Biochem, 1997. **245**(3): p. 557-63.
8. Cosson, B., et al., *Poly(A)-Binding Protein Acts in Translation Termination via Eukaryotic Release Factor 3 Interaction and Does Not Influence [PSI+] Propagation.* Molecular and Cellular Biology, 2002. **22**(10): p. 3301-3315.
9. Hodge, C.A., et al., *Rat8p/Dbp5p is a shuttling transport factor that interacts with Rat7p/Nup159p and Gle1p and suppresses the mRNA export defect of xpo1-1 cells.* EMBO J, 1999. **18**(20): p. 5778-88.

Table S2. Plasmids used in this study.

Plasmid number	Features	Source
pHK87	*CEN LEU2*	[1]
pHK88	*CEN URA3*	[1]
pHK607	*CEN lacZ-UAG-luc LEU2*	[2]
pHK608	*CEN lacZ-luc LEU2*	[2]
pHK629	*2µ DBP5 URA3*	[3]
pHK653	*CEN GFP-DBP5 URA3*	[3]
pHK666	*CEN GFP-DBP5 LEU2*	[4]
pHL693	*CEN* P_{Rat8}*rat8-2myc LEU2*	This Study
pHK698	*CEN Rpl25-GFP URA3*	[5]
pHK804	*CEN* P_{ADH1}*SUP35-GFP URA3*	This Study
pHK886	*2µ* P_{RLI1}*RLI1-HA TRP*	[6]
pHK887	*2µ* P_{RLI1}*RLI1-HA LEU2*	[6]
pHK1278	pET15b-*SUP45Δ1237-1311*	This study
pHK1280	pET28a-*SUP45*	This study
pHK1283	pGEX-4T1-*SUP35*	This study
pHK1288	pGEX-6P1	GE Healthcare
pHK1289	pGEX-6P1-*DBP5*	[4]
pHK1292	*2µ RLI1-GFP LEU2*	This study
pHK1323	*CEN* P_{ADH1}*3xMYC-GLE1 URA3*	This study
pHK1335	*CEN* P_{ADH1}*SUP45-GFP*	This study
pHK1397	CEN P_{uS3}uS3-GFP LEU2	This study
pHK1414	pGEX-6P1-RLI1	This study
pHK1474	*CEN RLI1-GFP TRP1*	This study
pHK1475	*CEN RLI1-GFP LEU2*	This study
pHK1617	pGEX 6P1-GST-eRF1	This study
pHK1618	pGEX 6P1-GST-eRF3ΔN165	This study

References for Table S2

1. Sikorski, R.S. and P. Hieter, *A system of shuttle vectors and yeast host strains designed for efficient manipulation of DNA in Saccharomyces cerevisiae.* Genetics, 1989. **122**(1): p. 19-27.
2. Stahl, G., et al., *Versatile vectors to study recoding: conservation of rules between yeast and mammalian cells.* Nucleic Acids Res, 1995. **23**(9): p. 1557-60.
3. Gross, T., et al., *The DEAD-box RNA helicase Dbp5 functions in translation termination.* Science, 2007. **315**(5812): p. 646-9.

4. Neumann, B., et al., *Nuclear Export of Pre-Ribosomal Subunits Requires Dbp5, but Not as an RNA-Helicase as for mRNA Export.* PLoS One, 2016. **11**(2): p. e0149571.
5. Gadal, O., et al., *Nuclear export of 60s ribosomal subunits depends on Xpo1p and requires a nuclear export sequence-containing factor, Nmd3p, that associates with the large subunit protein Rpl10p.* Mol Cell Biol, 2001. **21**(10): p. 3405-15.
6. Kispal, G., et al., *Biogenesis of cytosolic ribosomes requires the essential iron-sulphur protein Rli1p and mitochondria.* EMBO J, 2005. **24**(3): p. 589-98.

Table S3. Oligonucleotides used in this study.

Oligo number	Sequence	Target
HK1109	5’- cgtc**gaattc**ATGTCGGATTCAAACCAAG -3’	*SUP35* forward **(*Eco*RI site)**
HK1110	5’- ga**ctcgag**TTACTCGGCAATTTTAACAATTTTAC -3’	*SUP35* reverse **(*Xho*I site)**
HK1144	5’- gtc**catATG**GATAACGAGGTTGAAAAAAATATTG -3’	*SUP45* forward **(*Nde*I site)**
HK1146	5’- gt**ctcgag**ATGGATAACGAGGTTGAAAAAAATATTG -3’	*SUP45* forward **(*Xho*I site)**
HK1147	5’- gtcatca**ggatcc**TTAAACTTTGTAACGCAGCATG -3’	*SUP45Δ25 reverse* **(*Bam*HI site)**
HK1156	5’- gac**ctcgag**TTAAATGAAATCATAGTCGGATCC -3’	*SUP45* reverse **(*Xho*I site)**
HK1194	5’- ggcc**ctgcag**gaATGGCTAGCAAAGGAGAAG -3’	*GFP* forward **(*Pst*I site)**
HK1195	5’- ggcc**ctcgag**TTAGCAGCCGGATCC -3’	*GFP* reverse **(*Xho*I site)**
HK1398	5’- atc**ggatcc**ATGAGATTTGTGTTCGATGAG -3’	*GLE1* forward **(*Bam*HI site)**
HK1399	5’- tta**ggatcc**CTAAGGAGACATTTCCGG -3’	*GLE1* reverse **(*Bam*HI site)**
HK1613	5’- tctttattttcagggcgccATGAGATTTGTGTTCGATGAG -3’	*GLE1* forward
HK1614	5’- aagcttgtcgacggagctCTAAGGAGACATTTCCGGA -3’	*GLE1* reverse
HK2136	5'- tatagggcgaattg**gagct**CGACTATACGCTG -3'	P_{RLI1} forward **(*Sac*I site)**
HK2137	5'- cgggccccccctcgag**gtcgac**GTACCGGCCGCAAATTAAAG -3'	T_{CYC1} reverse **(*Sal*I site)**
HK2920	5'-gcggcaGGGCCCgcggccgctctagaactagtg-3'	uS3 forward **(*Apa*I site)**
HK2921	5'-ggtaccGGGCCCcccctc-3'	uS3 reverse **(*Apa*I site)**

Figure S1

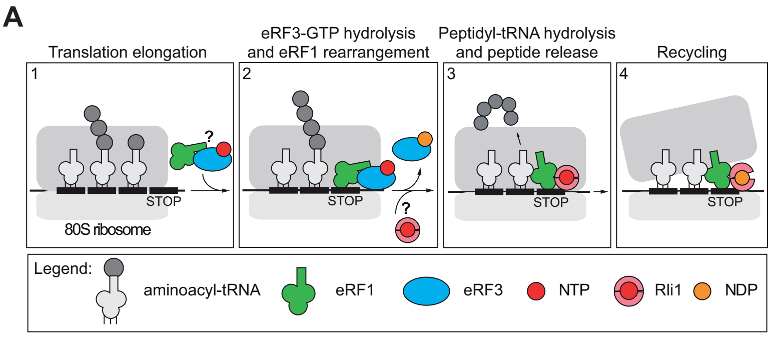
A
Translation elongation
eRF3-GTP hydrolysis
and eRF1 rearrangement
Peptidyl-tRNA hydrolysis
and peptide release
Recycling
1
2
3
4
?
STOP
80S ribosome
Legend:
aminoacyl-tRNA
eRF1
eRF3
NTP
Rli1
NDP

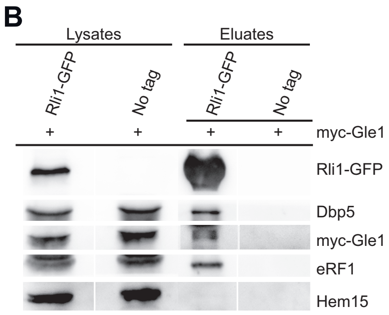
B
Lysates
Eluates
Rli1-GFP
No tag
Rli1-GFP
No tag
+
myc-Gle1
Rli1-GFP
Dbp5
myc-Gle1
eRF1
Hem15
